# ZnO nanoparticles modulate the ionic transport and voltage regulation of lysenin nanochannels

**DOI:** 10.1186/s12951-017-0327-9

**Published:** 2017-12-16

**Authors:** Sheenah L. Bryant, Josh E. Eixenberger, Steven Rossland, Holly Apsley, Connor Hoffmann, Nisha Shrestha, Michael McHugh, Alex Punnoose, Daniel Fologea

**Affiliations:** 1 Department of Physics, Boise State University, Boise, ID 83725 USA; 2 Biomolecular Sciences Graduate Program, Boise State University, Boise, ID 83725 USA; 3 Present Address: Department of Physics, University of Utah, Salt Lake City, UT 84112 USA; 4 Present Address: Department of Social Sciences, Yale-NUS College, Singapore, 138610 Singapore; 5 Present Address: Department of Chemical and Biological Engineering, Montana State University, Bozeman, MT 59717 USA

**Keywords:** ZnO, Nanoparticles, Lysenin, Ion transport, Electrophysiology, SnO_2_, Toxicity, Voltage gated channels, Ligand gated channels

## Abstract

**Background:**

The insufficient understanding of unintended biological impacts from nanomaterials (NMs) represents a serious impediment to their use for scientific, technological, and medical applications. While previous studies have focused on understanding nanotoxicity effects mostly resulting from cellular internalization, recent work indicates that NMs may interfere with transmembrane transport mechanisms, hence enabling contributions to nanotoxicity by affecting key biological activities dependent on transmembrane transport. In this line of inquiry, we investigated the effects of charged nanoparticles (NPs) on the transport properties of lysenin, a pore-forming toxin that shares fundamental features with ion channels such as regulation and high transport rate.

**Results:**

The macroscopic conductance of lysenin channels greatly diminished in the presence of cationic ZnO NPs. The inhibitory effects were asymmetrical relative to the direction of the electric field and addition site, suggesting electrostatic interactions between ZnO NPs and a binding site. Similar changes in the macroscopic conductance were observed when lysenin channels were reconstituted in neutral lipid membranes, implicating protein-NP interactions as the major contributor to the reduced transport capabilities. In contrast, no inhibitory effects were observed in the presence of anionic SnO_2_ NPs. Additionally, we demonstrate that inhibition of ion transport is not due to the dissolution of ZnO NPs and subsequent interactions of zinc ions with lysenin channels.

**Conclusion:**

We conclude that electrostatic interactions between positively charged ZnO NPs and negative charges within the lysenin channels are responsible for the inhibitory effects on the transport of ions. These interactions point to a potential mechanism of cytotoxicity, which may not require NP internalization.

**Electronic supplementary material:**

The online version of this article (10.1186/s12951-017-0327-9) contains supplementary material, which is available to authorized users.

## Background

The rapid development of certain nanomaterials (NMs) has led to their extensive use in many commercial applications including cosmetics, sporting goods, automotive parts, and electronics [1–4], while many others are under intense investigation for scientific, technological, and biomedical applications [5–9]. The large surface area to volume ratio of these materials yields novel physical and chemical properties that enable applications that are unachievable using micron-sized bulk material of identical composition. The scientific community has spent decades developing an understanding of NMs in order to control their fundamental physical and chemical properties. However, early investigations demonstrated that some of the same properties that make NMs attractive for multiple applications may cause unintended hazardous interactions with biological systems. Therefore, environmental and human exposure poses potentially significant risks [10], and this paradigm has led to intense investigations on the potential biological impact of NMs [11, 12]. While we have thus far attained a tremendous body of knowledge on end-point effects such as cytotoxicity, neurotoxicity, genotoxicity and oxidative stress [13–16], we lack a thorough understanding of the principles by which modulation of size, charge, composition, dissolution levels and surface chemistry affect the interaction of NMs with living cells.

ZnO nanoparticles (NPs) are considered to be one of the more toxic of the metal oxide NMs [17, 18]. Studies on ZnO NPs have demonstrated toxicity towards a large number of cell lines and model organisms, however, the mechanism of cytotoxicity is still under debate. Certain physicochemical properties, such as surface chemistry, dissolution potential, and their intrinsic ability to produce reactive oxygen species (ROS) have a strong impact on their cytotoxic effects [19–21]. Several studies have demonstrated that cytotoxicity stems from high dissolution rates, causing elevated levels of Zn^2+^ ions in cellular media that eventually disrupt homeostasis and leads to cell death [22, 23]. Other groups have suggested that their intrinsic ability to produce ROS (which may arise from surface defects, such as oxygen vacancies) is responsible for the high cytotoxic potential of ZnO NPs [24, 25]. In the same line, SnO_2_ NPs have been shown to inhibit kinetic growth and cytotoxicity towards certain cell lines and organisms [18, 26, 27], while other publications have demonstrated modest to no cytotoxic effects [28, 29]. Similar to other NPs, the crystal and hydrodynamic size of SnO_2_ NPs play an important role on their toxic effects, and smaller sizes have been shown to correlate with increased toxicity [27].

Our inability to correctly predict how physical and chemical properties relate to toxicity stems from the fact that biological systems are elaborate and structurally and functionally interconnected, making it very difficult to isolate distinct interactions responsible for cytotoxicity. Therefore, investigations utilizing a simplified model system that mimics the structure and function of a biological assembly can be an important step towards a more complete understanding of mechanisms of nanotoxicity. In these regards, we address how the directional flow of ions across lipid membranes containing specialized transmembrane ion transporters are affected by NPs. This work is motivated by the tremendous biological relevance of ionic transport for any living cell, and by the evidence that malfunctions of the mechanisms that control the transmembrane transport may have catastrophic consequences for cell functionality [30].

Among transmembrane transporters, voltage-regulated channels play key roles in fundamental cellular processes such as creating and maintaining electrochemical gradients, transmission of information, ion transport, signaling, and metabolism [31]. A salient feature of such transporters is the regulation of their activity by transmembrane electric fields interacting with voltage-sensing domains present in the channel’s structure [32]. The presence of charged domains in different regions of protein channels presents opportunities for electrostatic interactions with charged NPs, which may affect the transmembrane transport and functionality of the host cells.

Given the large variety of ion transporters in the cell membrane, isolating a particular one in a specific cell for relevant studies on transport modulation induced by NPs is not an easy task. Moreover, reconstitution of a particular ionic transporter in an artificial membrane system, although feasible, may require multiple, extensive and costly preparation steps. A simplified system featuring fundamental characteristics of ion channels may constitute an excellent model for investigating potential nanotoxicity effects originating from the disruption of transmembrane transport of ions. Therefore, we propose a simplified model that explores the effects of charged NPs on the transport of ions through lysenin channels inserted into an artificial bilayer lipid membrane (BLM).

Lysenin is a pore-forming protein extracted from the coelom of the earthworm *E. foetida,* which self-assembles as a large conductance nonameric pore (~ 3 nm) in artificial and natural lipid membranes containing sphingomyelin (SM) [33–35]. The recently deciphered crystal structure indicates large charged domains present within the channel [36, 37], thus presenting a strong potential for electrostatic interactions with charged NPs. The physiological role of lysenin is still obscure but the cytolytic and hemolytic activity is indicative of a pore-forming toxin [38]. Nonetheless, its relevance for nanotoxicity studies stem from several remarkable biophysical properties it shares with ion channels. Unlike many other pore-forming toxins and similar to voltage-gated ion channels, lysenin channels present asymmetrical voltage-induced gating [33, 39]. They adopt an open state at negative voltages, while positive voltages larger than ~ + 20 mV induce gating and closing [39, 40]. This salient feature is complimented by reversible ligand-induced gating, manifested as conformational changes in the presence of low concentrations of multivalent metal cations leading to channel closure [41, 42]. Once the multivalent cations bind and induce conformational changes, the channel adopts a sub-conducting or closed state [41, 42]. Another advantageous property of lysenin channels is that voltage and ligand-gating properties can be easily discriminated. This is achieved by reconstituting the channels in neutral lipid membranes which maintains the ligand-induced gating mechanism but renders lysenin unresponsive to the applied voltage [41, 42]. The high transport rate of lysenin channels yield large ionic currents which facilitate data recording and analysis. Lastly, lysenin channels are easily reconstituted in artificial membrane systems containing SM, are stable for extended time periods, and the monomer form of the protein is commercially available.

## Methods

### Chemicals and nanoparticles

Asolectine (Aso), cholesterol (Chol), SM (from Sigma-Aldrich) and diphytanoyl phosphatidylcholine (DiPhytPC, from Avanti Polar Lipids) were purchased as powders and dissolved in *n*-decane at a final concentration of 50 mg/mL. For the support electrolyte, NaCl (Fisher Scientific) was dissolved in nanopure water at a final concentration of 130 mM (if not otherwise indicated) and buffered with 20 mM 4-(2-hydroxyethyl)-1-piperazineethanesulfonic acid (HEPES) at pH = 7.2. ZnO and SnO_2_ NPs were synthesized using wet chemical methods as previously described [43, 44]. Briefly, for ZnO NP samples, the precursor zinc acetate dihydrate (Zn[CH_3_CO_2_]_2_·2H_2_O) was suspended in diethylene glycol. The solution was heated and nanopure water was added when the solution reached 80 °C. The temperature was then brought to and held at 150 °C for 90 min. The NPs were collected by centrifugation and subsequently washed with ethanol. For SnO_2_ NPs, sodium stannate (Na_2_[Sn(OH)_6_]) and urea were used as precursors with nanopure water as the solvent. The solution was heated to 90 °C and held for 90 min. The NPs were collected via centrifugation and subsequently washed with nanopure water. Characterizations were performed using X-ray diffraction (XRD) (Additional file 1: Fig. S1), transmission electron microscopy (TEM) (Additional file 1: Figs. S2, S3), zeta potential (ZP) measurements, dynamic light scattering (DLS) (Additional file 1: Fig. S4), X-ray photoelectron spectroscopy (XPS) (Additional file 1: Fig. S5) and Fourier-transform infrared spectroscopy (FTIR) (Additional file 1: Fig. S6). XPS confirmed sample purity and atomic concentrations for stoichiometric ratios. XRD was employed to ensure crystal phase purity and to obtain average crystalline size for both samples. XRD confirmed the expected hexagonal wurtzite crystal structure for ZnO and cassiterite for SnO_2_. The average crystal size for ZnO and SnO_2_ NPs was analyzed with Rietveld refinement using Materials Analysis Using Diffraction (MAUD) software and estimated at 8.3 ± 2 and 4.3 ± 0.04 nm respectively. A JEOL JEM-2100 HR analytical TEM was used to confirm spherical morphology and average crystal sizes. FTIR spectra was collected using a Bruker Tensor 247 spectrometer and FTIR pellets were produced by first grinding 1.6 mg of each NP sample with 0.200 g of spectroscopic grade KBr. The ground powder mixture was then pressed with 8 tons of pressure for 3 min and pellets were analyzed after removing the KBr background. Zeta potential and DLS measurements were performed, after dispersing the powders in nanopure water at a concentration of 1 mg/mL, using a Malvern Zetasizer NanoZS. ZnO NP clusters had an average hydrodynamic size (HDS) of 276 nm and average ZP of + 32 mV, whereas SnO_2_ NP clusters average HDS was 176 nm with an average ZP of − 42.0 mV.

### Bilayer lipid membrane setup

The experimental setup employed the use of a planar BLM chamber consisting of two polytetrafluoroethylene (PTFE) reservoirs separated by a thin (~ 120 μm) PTFE film that had been pierced with an electric spark to create a circular hole of ~ 70 µm diameter [45, 46]. The reservoirs were filled with 1 mL buffered electrolyte and connected via two Ag/AgCl electrodes inserted in the solution to an Axopatch 200B amplifier (molecular devices). The amplified analog signal fed the DigiData 1440A digitizer (molecular devices) which provided the digital signal for visualization, recording, and further analysis. Continuous stirring of the solutions in the BLM chamber was assured by a low-noise magnetic stirrer (Warner instruments). All the experiments were performed in voltage-clamp mode upon manual or automatic voltage stimulation. The signal recorded during various voltage stimulations was further analyzed with ClampFit 10.6.2.2 (Molecular Devices) and Origin 8.5.1 (Origin Lab) software packages.

### Experimental procedure

Lipid membrane preparation was performed by “painting” the hole in the PTFE film with small amounts of lipid mixtures composed of 4 mg Aso or DiPhytPC, 2 mg Chol, and 2 mg SM dissolved in ~ 400 μL *n*-decane [46, 47]. The successful creation of the BLM was indicated by measuring the capacitance in response to an applied triangular voltage stimulation, while achievement of a seal resistance larger than 1000 GΩ was assessed by measuring the leakage current in response to a DC voltage stimulation (100 mV). Channel insertion was performed by adding the lysenin monomer (from Sigma-Aldrich, 0.3 nM final concentration) to the ground (*cis*) reservoir under continuous stirring and at − 60 mV bias potential applied to the *trans* (headstage) reservoir. The application of a negative voltage was required to prevent the voltage-induced gating which manifests at positive transmembrane potentials [33, 39, 40]. After the insertion process was completed, as indicated by a steady state value of the open current, an extensive flushing of the *cis* reservoir with lysenin-free electrolyte was performed to remove the bulk monomer and prevent additional insertions. To avoid potential changes in the lysenin functionality originating in congestion effects [48], the total number of channels inserted into the membranes was limited to ~ 1000. To facilitate quantitative comparison of the influence of NPs on the transport properties of lysenin channels in parallel experiments comprising different numbers of inserted channels, we used the relative changes in the macroscopic conductance (G_r_ = G/G_0_) for data plotting, where G is the conductance after addition of NPs and G_0_ is the conductance before addition. In order to avoid premature dissolution and/or aggregation, the NPs (powder form) were dispersed by sonication for 5 min in the support electrolyte solution in a sonication bath before each addition to the reservoirs.

## Results and discussion

**Fig. 1:**
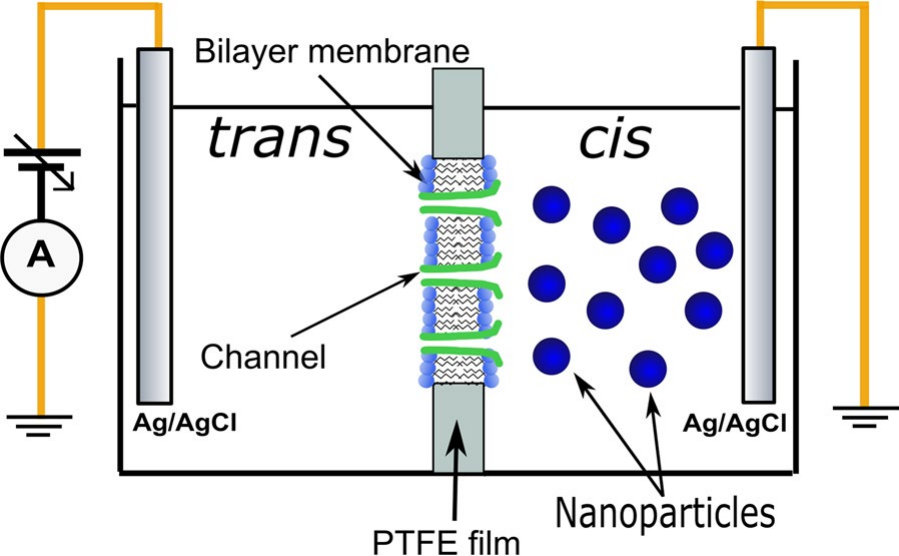
The experimental setup comprises lysenin channels reconstituted into planar lipid bilayer membranes. The modulation of ionic transport and regulation by ZnO NPs is assessed in classic voltage-clamp experiments

**Fig. 2:**
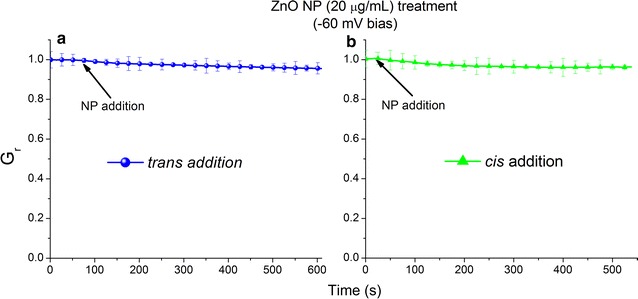
ZnO NPs do not alter the ionic conductance of lysenin channels when biased by a − 60 mV transmembrane voltage. Addition of ZnO NPs to either *trans* (**a**) or *cis* (**b**) reservoirs induces only negligible changes of the macroscopic conductance. The experimental values are reported as mean ± SD, n = 3. All the data points represent experimental values but some symbols have been removed for improved visibility

Once a steady state current through the population of lysenin channels was achieved, the NPs were introduced into either side of the chamber with both negative and positive voltages applied across the membrane to assess their effect on the macroscopic conductance (see Fig. 1 for a schematic of the setup). The addition of ZnO NPs (20 µg/mL final concentration) to either side of the membrane containing lysenin channels, when biased by − 60 mV, yielded only a modest decrease of the macroscopic conductance, i.e. a few percent, irrespective of the side of addition (Fig. 2). This slight decrease in the conductance suggests a minimal influence of ZnO NPs on the lysenin channels’ ability to transport ions in these particular experimental conditions.

To explain the small reduction in conductance, one may hypothesize several different mechanisms such as ligand gating induced by small amounts of Zn^2+^ ions provided from low NP dissolution, ligand gating induced by NP binding to a specific binding site, or physical occlusion by transient NP attachment to the opening of the nanopore. Past investigations show a dramatic yet reversible decrease of the macroscopic conductance of lysenin channels in the presence of low concentrations of multivalent cations [41, 42], indicative of strong interactions with lysenin channels. Those interactions have been elucidated in single-channel experiments, which provide evidence of gating, i.e. transition from the open state to a sub-conducting or closed state [41, 42]. To explain lysenin’s reversible gating in the presence of multivalent cations, it is assumed that the channel’s structure contains at least one negatively-charged binding site with high affinity for cations, which triggers gating upon binding. A potential leakage of Zn^2+^ ions from NPs may affect the macroscopic conductance of lysenin channels, as observed. In addition, if exposed this binding site could electrostatically interact with cationic NPs and yield a significant decrease in conductance either by induced gating or physical occlusion of the conducting pathway. However, such strong effects were not observed in the above experimental conditions, which prompted us to look closer to the lysenin’s structure for alternative explanations. The assembled lysenin channel shows the presence of multiple anionic domains [36, 37, 49], hence presenting opportunities for physical occlusions of the channels through electrostatic interactions even in the absence of gating. We may account for the weak conductance inhibition by considering the position of a binding site and the orientation of the external electric field. A deep-buried binding site would be inaccessible from either side to NPs larger than the channel’s diameter (~ 3 nm), which is mostly the case in our investigations. Nanoparticle interaction with a binding site present at the *trans* opening of the channel would be prevented at − 60 mV by the electric field orientation. Although the electric field in the bulk is very low, its amplitude increases substantially when approaching the channel opening (fringe effects), therefore keeping the NPs far from a binding site located at the *cis* opening. The same electric field will drive the NPs added to the *cis* side towards the membrane but the lack of changes in macroscopic conductance suggest the absence of a binding site at this location. The hypothesis of an exposed binding site at the *trans* opening was further sustained in similar experiments comprising *trans* NP addition and no transmembrane voltage; in such experimental conditions, a marked decrease of the macroscopic conductance was observed at − 60 mV after 2 h of NP incubation in the absence of a bias potential (data not shown). However, this result could be an artifact originating from dissolution during the prolonged NP exposure to the electrolyte solution.

**Fig. 3:**
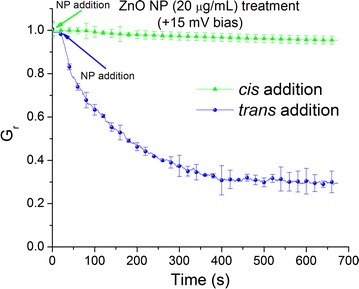
Interactions between lysenin channels and ZnO NPs at + 15 mV bias potential. *Cis* addition (green) of ZnO NPs yield minor changes in the macroscopic conductance. In contrast, *trans* addition (blue) elicits a significant decrease of the macroscopic conductance by ~ 70%. The experimental values are reported as mean ± SD, n = 3. All the data points represent experimental values but some symbols have been removed for improved visibility

To identify if the elusive binding site is located either deep within the channel or at the *trans* side, we performed the experiments under positive bias potentials (Fig. 3). After the channel insertion process, the influence of ZnO NPs was assessed in experiments comprising of *cis* or *trans* addition and opposite orientations of the electric field. Lysenin channels are voltage-gated at positive voltages greater than ~ + 20 mV but are stable in the open state for extended time periods as long as the applied voltage is less than this critical value [33, 39]. Interestingly, addition of ZnO NPs to the *trans* side under positive biasing (+ 15 mV to prevent voltage gating) induced a rapid and sustained decrease of the macroscopic conductance (Fig. 3), while *cis* addition elicited only a weak response in otherwise similar conditions. Consequently, we concluded that the electric field plays a major role in preventing ZnO NPs accumulation near the membrane when biased by − 60 mV. However, in the absence of an electric field or when positive voltages are applied, ZnO NPs may interact with a binding site situated at the *trans* opening of the channel.

**Fig. 4:**
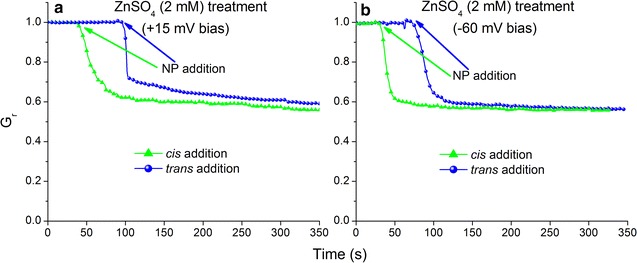
Zn^2+^ ions decrease the macroscopic conductance of lysenin channels irrespective of bias potential and site of addition. At + 15 mV transmembrane voltage (**a**), Zn^2+^ addition to either the *cis* or *trans* reservoir reduces the macroscopic conductance by ~ 40%. Similar decreases are recorded upon Zn^2+^ interactions with lysenin channels biased by − 60 mV (**b**). The presented data represents a typical run for each experiment

Dissolution of ZnO NPs can result in high extracellular Zn^2+^ concentrations which have been proposed as one of the main mechanisms of ZnO NPs cytotoxic effects [22, 23, 50]. Zinc ions inhibit the macroscopic conductance of lysenin channels by a ligand-induced gating mechanism [41, 42]. Due to the high sensitivity of lysenin channels to Zn^2+^, dissolution may explain the observed inhibition of conductance upon exposure to ZnO NPs. To eliminate such potential experimental artifacts, we performed investigations in similar conditions but added Zn^2+^ ions (ZnSO_4_; 2 mM final concentration) to the reservoirs instead of ZnO NPs. Addition of Zn^2+^ to either side, biased by − 60 mV, yielded a sudden decrease of the macroscopic conductance in agreement with previous reports (Fig. 4) [41, 42]. Addition of the same amount of Zn^2+^ to a similar BLM containing lysenin channels and biased by + 15 mV (to prevent voltage-induced gating) yielded a similar relative decrease of the macroscopic open current (Fig. 4). If conductance inhibition elicited by ZnO NPs had been induced by the Zn^2+^ ions dissipating from the NPs, then addition to either side would have displayed a similar pattern of conductance inhibition. However, addition of Zn^2+^ ions yielded fundamentally different results compared with the experiments involving ZnO NPs. Zn^2+^ ions affected the macroscopic conductance irrespective of the side of addition and direction of the electric field, while the inhibitory activity of ZnO NPs depended on both these experimental parameters. The total concentration of ZnO NPs was only 20 μg/mL (corresponding to ~ 0.25 mM Zn^2+^ ions) and resulted in a 70% decrease in the macroscopic conductance. In order to obtain an approximate decrease of only 45% in conductance measurements with Zn^2+^ ions, the experiment employed a final concentration of 2.0 mM. Assuming complete dissolution of ZnO NPs, this would correlate to approximately eight times the amount of Zn^2+^ ions from ZnSO_4_ in the solution. To further eliminate the possibility that the Zn^2+^ ions contributed to the observed conductance inhibition, experiments with ZnO NPs were carried out in the presence of the strong Zn^2+^ chelator EDTA. EDTA (10 mM) was added to the solutions prior to nanoparticle addition, thus effectively preventing any interactions of the free zinc ions from the NPs with lysenin channels. These experiments yielded almost identical decreases in the macroscopic conductance when compared with ZnO NPs with no EDTA (Additional file 1: Fig. S7). Our results clearly indicate that the conductance inhibition elicited by ZnO NPs was not a consequence of Zn^2+^ ions from dissolution. These experiments revealed that the extent of the conductance inhibition depended on both the orientation of the lysenin channels and the electric field relative to the site of ZnO NP addition. The observed conductance inhibition may originate from local accumulation of NPs by electrophoretic effects, specific interactions with the membrane itself, or preferential interactions with binding sites of lysenin.

**Fig. 5:**
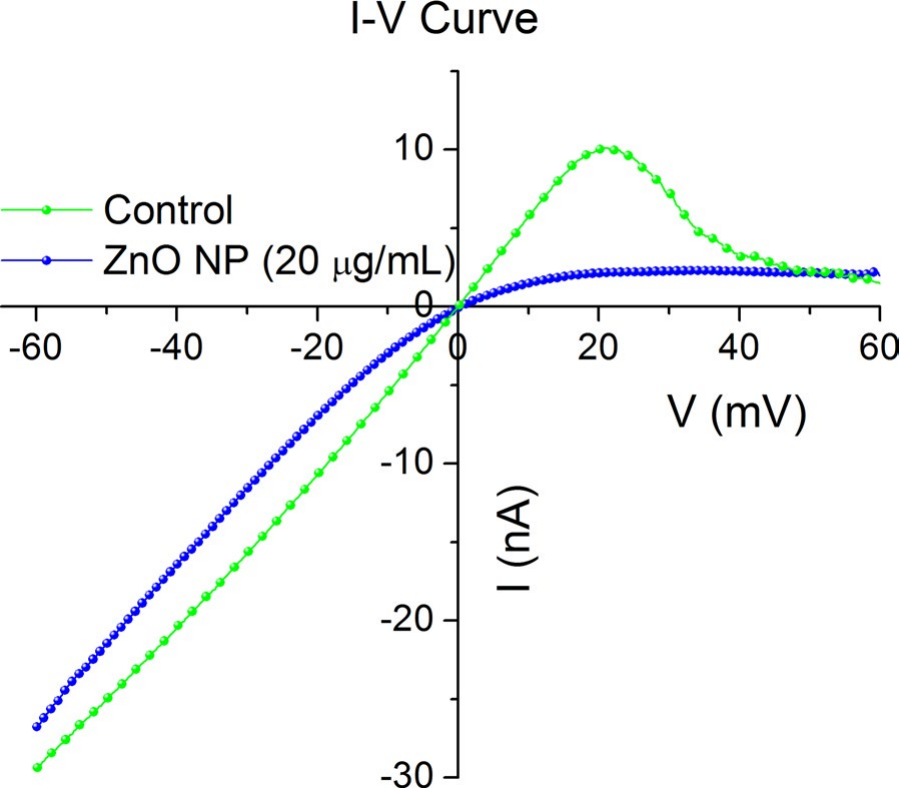
Effects of ZnO NPs on lysenin voltage-induced gating. In the absence of NPs, lysenin channels begin to close at transmembrane potentials greater than 20 mV (green curve). ZnO NPs (20 µg/mL final concentration) almost completely abrogate the conductance in the positive voltage range (blue curve) and indicate a strong interaction with the lysenin channels. All points on the curves are experimental data and symbols have been added for discrimination. The presented data represents a typical run for each experiment

Next, we asked whether or not exposure to ZnO NPs changes the voltage-induced gating profile. To answer this question, the voltage-induced gating of lysenin channels was assessed from the I–V plot recorded in the range − 60 to + 60 mV (Fig. 5) at a voltage rate of 0.2 mV/s with and without the addition of ZnO NPs. The macroscopic current recorded in absence of NPs (Fig. 5) featured the well-known characteristics of voltage-induced gating, i.e. a linear behavior in the negative voltage range, indicative of the absence of gating, and a non-linear behavior at positive voltages higher than + 20 mV, indicative of channel closure [33, 39, 40]. A typical feature of the macroscopic current recorded at positive voltages is the transition from high current to low current through a dynamic negative resistance region [39, 40]. The macroscopic currents recorded in the same voltage range after addition of ZnO NPs (20 µg/mL final concentration) to the *trans* side of the membrane yielded a fundamentally different I–V plot (Fig. 5). The addition of ZnO NPs elicited a slight decrease in the open current recorded in the negative voltage range, however, the I–V characteristic preserved quasi-linearity between − 60 and − 20 mV. Once the voltage approached neutral values, the macroscopic current greatly deviated from the control I–V plot and the ionic transport capabilities of lysenin channels were strongly diminished in the presence of ZnO NPs. Interestingly, the macroscopic conductance started to decrease at small negative voltages, as indicated by the diminished slope of the I–V plot. This is consistent with the hypothesis that the fringe effect of the electric field prevents the NPs from interacting with the binding site. The magnitude of the electric field decreases with decreasing applied voltage and the weak electrophoretic force, although opposed, is not sufficient to prevent interactions with the binding site and channel conductance modulation. The consistently lower macroscopic currents indicated that addition of ZnO NPs induced severe channel conductance inhibition as demonstrated by the large decrease of the macroscopic current at any positive voltage. At positive voltages, the currents recorded in the presence of ZnO NPs were consistently lower than the currents recorded in the absence of ZnO NPs up to ~ + 40 mV, after which the recorded currents were similar to the control when the channels are in a closed state.

**Fig. 6:**
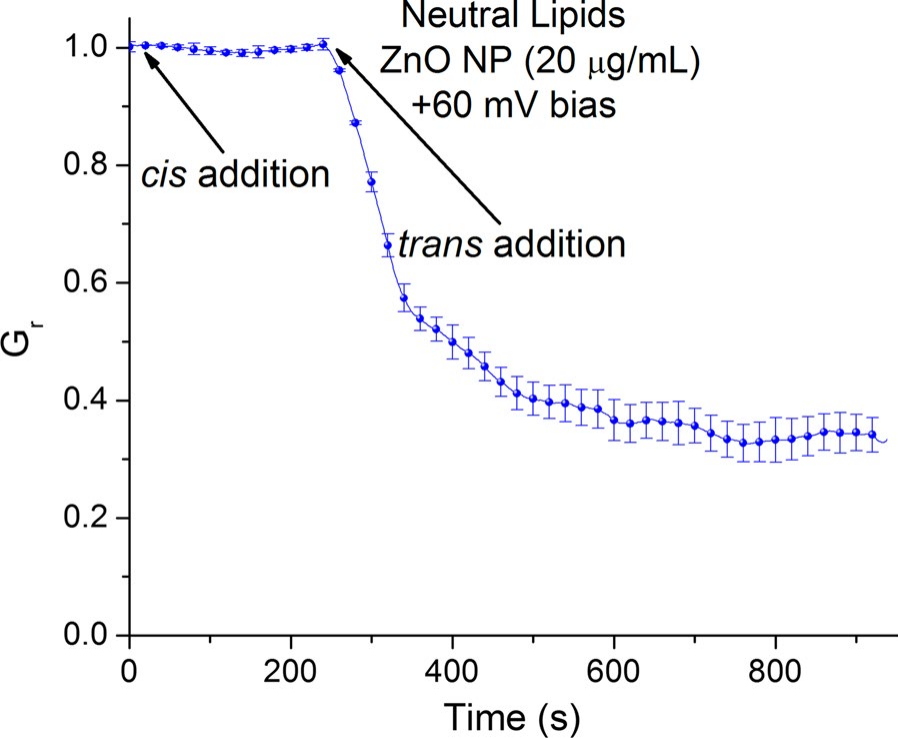
Lysenin channels reconstituted in neutral lipid membranes interact with ZnO NPs at + 60 mV transmembrane voltage. *Cis* addition of ZnO NPs elicits no changes in the macroscopic conductance. In contrast, ZnO NPs added to the *trans* reservoir interact with lysenin channels and significantly diminish their ionic transport capabilities. The experimental values are reported as mean ± SD, n = 3. All the data points represent experimental values but some symbols have been removed for improved visibility

This experiment demonstrated that ZnO NPs affect the macroscopic conductance of lysenin channels in a voltage-dependent manner but it does not offer a complete mechanistic description. The significant changes in the I–V curve after addition of ZnO NPs potentially stem from multiple mechanisms. Experimental evidence and theoretical modeling have demonstrated that electrostatic interactions between membrane components and NPs are key factors that contribute to toxicity and the ability of NPs to internalize into cells [51–53]. Our experiments comprised a simple system consisting of lysenin channels inserted into an artificial BLM composed of charged lipids. We assumed that the conductance of lysenin channels was affected by interactions between the protein channels and NPs but we could not exclude interactions between the charged lipids and ZnO NPs as a source of conductance modulation. The Aso lipid mixture used for BLM preparation contains several anionic components that may interact electrostatically with voluminous cationic NPs unable to penetrate the lumen, which would lead to channel conductance modulation. To elucidate the potential role played by the charged lipids, we performed experiments by replacing Aso with neutral DiPhytPC. The use of neutral lipids abolishes the voltage-induced gating at positive voltages while preserving the ligand-induced gating observed in the presence of multivalent cations [41, 42]. Addition of ZnO NPs to the *cis* side of a neutral membrane containing lysenin channels and biased by + 60 mV elicited no change in the macroscopic conductance (Fig. 6). However, addition of ZnO NPs to the *trans* side of the same membrane, biased by an identical positive voltage, yielded a massive decrease in conductance similar to the results obtained using charged lipids (Fig. 6). The non-symmetrical response and preservation of the inhibitory capabilities of ZnO NPs recorded for the neutral BLM suggest that the inhibition mechanism excludes electrostatic interactions between NPs and lipids. The interaction between lysenin channels and ZnO NPs is therefore likely responsible for the observed inhibitory activity.

**Fig. 7:**
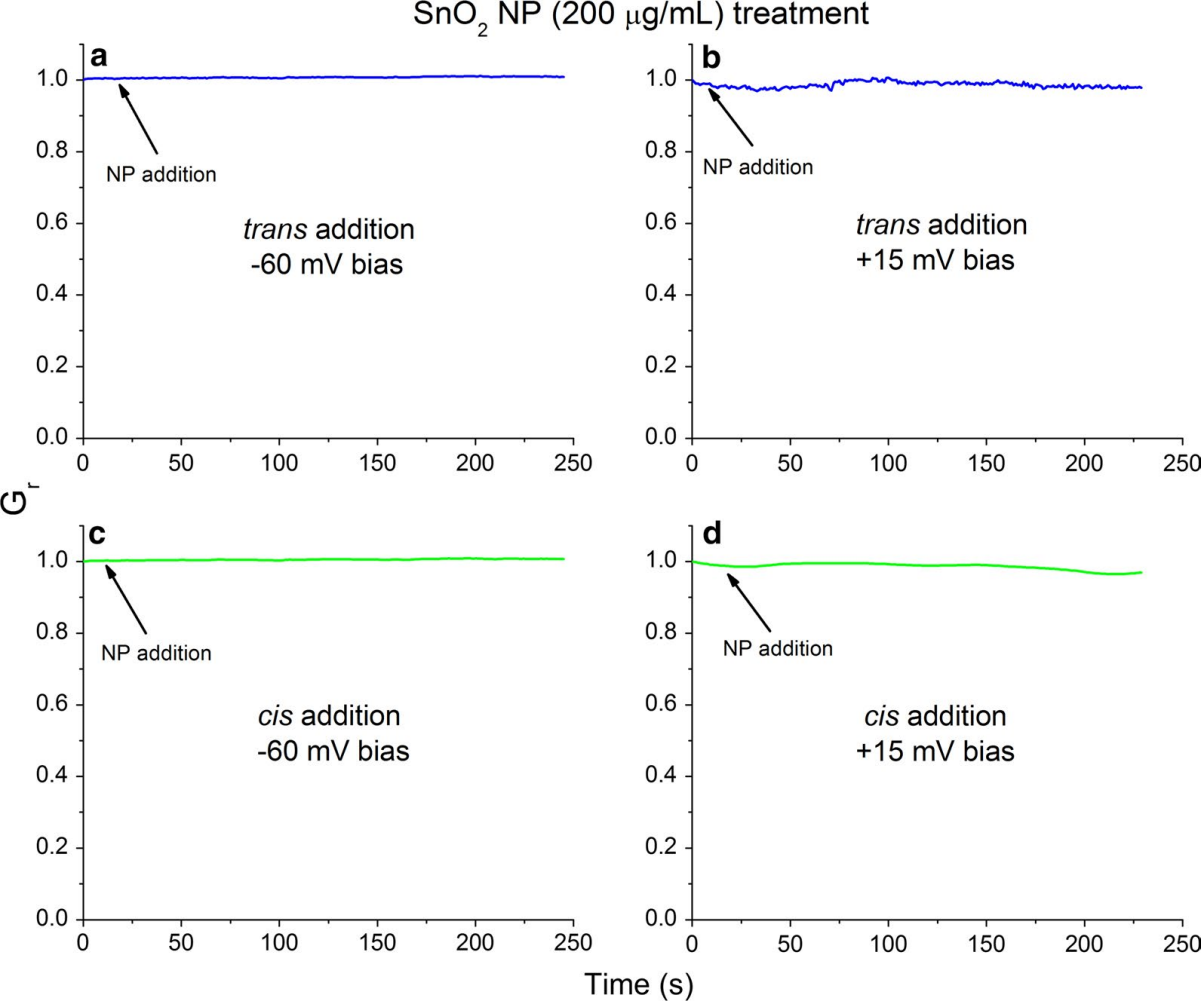
Interactions between anionic SnO_2_ NPs and lysenin channels reconstituted into a planar bilayer lipid membrane. Addition of SnO_2_ NPs to the *trans* reservoir at − 60 mV (**a**) and + 15 mV (**b**) indicates insignificant changes of the macroscopic conductance. Similarly, SnO_2_ NP addition to the *cis* reservoir at − 60 mV (**c**) and + 15 mV (**d**) yields negligible changes in the ionic transport capabilities. The presented data represents a typical run for each experiment

We have shown that lysenin channels interact with positively charged ZnO NPs but have not yet demonstrated the electrostatic nature of those interactions. Therefore, we asked whether or not any NPs electrophoretically driven towards a specific or non-specific yet accessible binding site would interact with lysenin channels and inhibit their conductance. In this respect, we performed conductance experiments by replacing positively charged ZnO NPs with negatively charged SnO_2_ NPs (− 42 mV ZP). Irrespective of the applied voltage and the addition site, SnO_2_ NPs did not affect the macroscopic conductance of lysenin channels (Fig. 7). In order to try to elicit interactions with SnO_2_ NPs, 200 μg/mL (final concentration) of SnO_2_ NPs were used, which is 10× the concentration of ZnO NPs that induced rapid decreases in the macroscopic conductance (Fig. 3). The crystal and hydrodynamic sizes of SnO_2_ NPs used in this experiment were much smaller than ZnO NPs, suggesting that SnO_2_ NPs would be better suited to inhibit conductance by physical occlusion. The absolute magnitude of the ZP for SnO_2_ NPs was also larger than ZnO NPs, further strengthening the hypothesis of a mechanism that requires strong electrostatic interactions between cationic ZnO NPs and an anionic domain present at the *trans* side of the lysenin channel to induce conductance inhibition. Also, to further support the hypothesis that electrostatic interactions between the lysenin channels and ZnO NPs initiate a decrease in conductance, we investigated the effects of electrostatic screening induced by an increased ion concentration in the bulk electrolyte solutions. Addition of 20 µg/mL ZnO NPs to the *trans* side of the bilayer containing lysenin channels in 500 mM NaCl and under positive bias reduced the conductance by ~ 15% (Additional file 1: Fig. S8), which is much smaller than what we observed at 130 mM NaCl concentration (~ 70%, Fig. 2). In addition, the time required to reach equilibrium increased to more than 2500 s, indicating that ionic screening weakened the interactions between NPs and lysenin channels, and supporting the hypothesis that electrostatic interactions are at the origin of the observed changes in conductance. However, we may not eliminate potential artifacts arising from the effects of screening on the ZnO NPs. At high salt concentration, screening may accelerate NP aggregation, which is what we observed when attempting to further increase the ionic concentration of the bulk electrolyte solutions. The ZnO NPs rapidly aggregated into large clusters at the bottom of the vials in a matter of minutes, which prevented further experimentation in high ionic strength conditions.

A few assumptions can be proposed about the mechanism responsible for the observed decrease in macroscopic conductance elicited by addition of ZnO NPs. Electrostatic interactions may bring ZnO NPs close enough to the channels such that the resulting physical blockage reduces the individual currents. In such case, an opposite electric field of appropriate magnitude may drive the NPs away from the binding site therefore unblocking the channels. Our attempts to apply higher voltages across the BLM and to force the unblocking were not successful. However, it is possible for the binding site to have a relatively strong affinity for charged ZnO NPs and consequently the force required to remove the NPs from the binding sight may require much higher electric fields. Unfortunately, such experiments are very difficult to achieve as the BLM is prone to disruption at high transmembrane voltages.

Another potential inhibition mechanism mimics ligand-induced gating. It has been established that lysenin channels interact with multivalent cations and undergo conformational transitions that force the channel into closed or sub-conducting states [41, 42]. This ligand-induced gating mechanism relies on electrostatic interactions between cations and one or more binding sites but ionic current blockage stems from the induced gating. It is possible that charged ZnO NPs interact electrostatically with one or more binding sites, yet not necessarily the same one(s) involved in the ligand-induced gating and would force the channels to adopt a sub-conducting or a closed state. Lastly, defects on the surface of ZnO NPs such as oxygen vacancies have been shown to correlate with ROS production [24, 54]. Since the electrostatic interactions induce close contact of ZnO NPs with the channels, the highly reactive surface of ZnO NPs may interact with cysteine and methionine residues in their structure which may alter channel functionality and conduction similar to reports of oxidation of cysteine residues in Ca^2+^/K^+^ channels [55, 56].

## Conclusions

Our work demonstrates that the transport properties of lysenin channels change significantly in the presence of cationic ZnO NPs. The modulation of the transport properties by NPs is strongly dependent on the net charge, and the orientation of the electric field and channel with respect to the NPs. There is little doubt that the primary interaction between NPs and lysenin channels is electrostatic. Nonetheless, the simplicity of the experimental system investigated here does not necessarily warrant biological interpolation to other protein channels interacting with NPs, not even ZnO. In complex biological environments, the binding of various functional groups on the NP surface may significantly alter their ability to interact with membrane components irrespective of the surface charge of the pristine nanomaterial. Given the aggregation tendency of the investigated NPs, we may not exclude aggregation at the membrane surface as being at the origin of conductance changes. Even the neutral lipids used for our investigations present a dipole moment that may initiate NP binding; further NP aggregation at these binding sites may impede the ionic flow by physical occlusion or by introducing supplementary electrostatic energy barriers for ions. However, if an induced dipole moment that initiated binding of NPs to lipids occurred, then SnO_2_ NPs should have yielded a similar response due to their higher net charge. Nonetheless, dipole–charge interactions have a much smaller magnitude than the charge–charge ones, and we did not observe such effects when using neutral lipids. In spite of these shortcomings, an important conclusion of this report pertains to the potential ability of NPs to interact with transmembrane transporters without the need of internalization. Many previous studies assume that cytotoxic effects of NPs are due to translocation of NPs into the cytosol by various transport mechanisms and/or dissolution of the NPs, disrupting homeostasis and interfering with vital cellular processes. Our work suggests that NPs may tamper with ionic transport mechanisms by basic electrostatic interactions. Given the physiological relevance of controlled transmembrane transport, such alterations may have catastrophic effects for cells. While this observation is generally valid for any cell, it may prove extremely helpful for understanding the potential neuro-toxic effects of NPs [57]. The physiology of the neural cell is based on the transport properties and regulation of voltage-gated ion channels, which are transmembrane structures with multiple charged domains that may interact electrostatically with NPs. Changes in the voltage-induced gating mechanism or blockage of ionic transport induced by NPs [57] may dramatically affect the correct functionality of the nerve cell. Such interactions may explain why certain NPs specifically alter the individual currents through specific channels while the transport properties of other channels are not affected by various NPs [58, 59]. The local distribution of charge within the structure of several ion channels is currently known so it may be possible to predict potential toxic effects based on interactions with charged NPs, or to design NPs intended to alter the activity of transmembrane transporters.

This foray into deciphering the effects of NPs on the transmembrane transport of ions indicates alterations in the transporters’ functionality as a potential mechanism of cytotoxicity. A previous study shows that ZnO NPs may induce neuronal cytotoxicity and genotoxicity in the absence of internalization or free Zn^2+^ ions released from the NPs [60]. Future experiments will shed more light on intimate mechanistic details and the role that electrostatic interactions play in modulating the biological activity of protein channels.

## Additional file

**Additional file 1.** Additional material that includes complementary figures of material characterization (**Figs. S1**–**S6**: XRD, TEM, DLS, XPS, and FTIR for ZnO and SnO_2_ NPs), changes in relative macroscopic conductance of lysenin channels in the presence of ZnO NPs and EDTA (**Fig. S7**), and conductance inhibition at high ionic strength (**Fig. S8**).

## Abbreviations

NMs: nanomaterials
NPs: nanoparticles
ROS: reactive oxygen species
BLM: bilayer lipid membrane
SM: sphingomyelin
Aso: asolectine
Chol: cholesterol
DiPhytPC: diphytanoyl phosphatidylcholine
HEPES: 4-(2-hydroxyethyl)-1-piperazineethanesulfonic acid
XRD: X-ray diffraction
TEM: transmission electron microscopy
ZP: zeta potential
DLS: dynamic light scattering
XPS: X-ray photoelectron spectroscopy
HDS: hydrodynamic size
PTFE: polytetrafluoroethylene

## References

[1] Zhang YY, Leu YR, Aitken RJ, Riediker M (2015). Inventory of engineered nanoparticle-containing consumer products available in the Singapore retail market and likelihood of release into the aquatic environment. Int J Environ Res Public Health.

[2] Sonneville-Aubrun O, Simonnet JT, L’Alloret F (2004). Nanoemulsions: a new vehicle for skincare products. Adv Colloid Interface Sci.

[3] Magdassi S, Grouchko M, Kamyshny A (2010). Copper nanoparticles for printed electronics: routes towards achieving oxidation stability. Materials.

[4] Karnaushenko D, Makarov D, Stober M, Karnaushenko DD, Baunack S, Schmidt OG (2015). High-performance magnetic sensorics for printable and flexible electronics. Adv Mater.

[5] Archana PS, Jose R, Vijila C, Ramakrishna S (2009). Improved electron diffusion coefficient in electrospun TiO_2_ nanowires. J Phys Chem C.

[6] McNamara K, Tofail SAM (2017). Nanoparticles in biomedical applications. Adv Phys X.

[7] Nune SK, Gunda P, Thallapally PK, Lin YY, Forrest ML, Berkland CJ (2009). Nanoparticles for biomedical imaging. Expert Opin Drug Deliv.

[8] Hu CMJ, Zhang L, Aryal S, Cheung C, Fang RH, Zhang LF (2011). Erythrocyte membrane-camouflaged polymeric nanoparticles as a biomimetic delivery platform. Proc Natl Acad Sci USA.

[9] Hsieh CT, Lin JS, Chen YF, Lin CY, Li WY (2014). Graphene sheets anchored with ZnO nanocrystals as electrode materials for electrochemical capacitors. Mater Chem Phys.

[10] Keller AA, McFerran S, Lazareva A, Suh S (2013). Global life cycle releases of engineered nanomaterials. J Nanopart Res.

[11] Ahamed M, AlSalhi MS, Siddiqui MKJ (2010). Silver nanoparticle applications and human health. Clin Chim Acta.

[12] Teow Y, Asharani PV, Hande MP, Valiyaveettil S (2011). Health impact and safety of engineered nanomaterials. Chem Commun.

[13] Hanley C, Layne J, Punnoose A, Reddy KM, Coombs I, Coombs A, Feris K, Wingett D (2008). Preferential killing of cancer cells and activated human T cells using ZnO nanoparticles. Nanotechnology.

[14] Hu YL, Gao JQ (2010). Potential neurotoxicity of nanoparticles. Int J Pharm.

[15] Chen T, Yan J, Li Y (2014). Genotoxicity of titanium dioxide nanoparticles. J Food Drug Anal.

[16] Kumar A, Pandey AK, Singh SS, Shanker R, Dhawan A (2011). Engineered ZnO and TiO_2_ nanoparticles induce oxidative stress and DNA damage leading to reduced viability of *Escherichia coli*. Free Radical Biol Med.

[17] Ivask A, Titma T, Visnapuu M, Vija H, Kakinen A, Sihtmae M, Pokhrel S, Madler L, Heinlaan M, Kisand V (2015). Toxicity of 11 metal oxide nanoparticles to three mammalian cell types in vitro. Curr Top Med Chem.

[18] Seabra AB, Duran N (2015). Nanotoxicology of metal oxide nanoparticles. Metals.

[19] Punnoose A, Dodge K, Rasmussen JW, Chess J, Wingett D, Anders C (2014). Cytotoxicity of ZnO nanoparticles can be tailored by modifying their surface structure: a green chemistry approach for safer nanomaterials. ACS Sustain Chem Eng.

[20] Sharma V, Anderson D, Dhawan A (2012). Zinc oxide nanoparticles induce oxidative DNA damage and ROS-triggered mitochondria mediated apoptosis in human liver cells (HepG2). Apoptosis.

[21] Xia T, Kovochich M, Liong M, Madler L, Gilbert B, Shi HB, Yeh JI, Zink JI, Nel AE (2008). Comparison of the mechanism of toxicity of zinc oxide and cerium oxide nanoparticles based on dissolution and oxidative stress properties. ACS Nano.

[22] Buerki-Thurnherr T, Xiao LS, Diener L, Arslan O, Hirsch C, Maeder-Althaus X, Grieder K, Wampfler B, Mathur S, Wick P, Krug HF (2013). In vitro mechanistic study towards a better understanding of ZnO nanoparticle toxicity. Nanotoxicology.

[23] Kao YY, Chen YC, Cheng TJ, Chiung YM, Liu PS (2012). Zinc Oxide nanoparticles interfere with zinc ion homeostasis to cause cytotoxicity. Toxicol Sci.

[24] Yang Q, Lin TS, Burton C, Park SH, Ma Y (2016). Physicochemical insights of irradiation-enhanced hydroxyl radical generation from ZnO nanoparticles. Toxicol Res.

[25] Akhtar MJ, Ahamed M, Kumar S, Khan MM, Ahmad J, Alrokayan SA (2012). Zinc oxide nanoparticles selectively induce apoptosis in human cancer cells through reactive oxygen species. Int J Nanomed.

[26] Roopan SM, Kumar SHS, Madhumitha G, Suthindhiran K (2015). Biogenic-production of SnO_2_ nanoparticles and its cytotoxic effect against hepatocellular carcinoma cell line (HepG2). Appl Biochem Biotechnol.

[27] Chavez-Calderon A, Paraguay-Delgado F, Orrantia-Borunda E, Luna-Velasco A (2016). Size effect of SnO_2_ nanoparticles on bacteria toxicity and their membrane damage. Chemosphere.

[28] Krysanov EY, Pavlov DS, Demidova TB, Dgebuadze YY (2010). Effect of nanoparticles on aquatic organisms. Biol Bull.

[29] Ai J, Biazar E, Jafarpour M, Montazeri M, Majdi A, Aminifard S, Zafari M, Akbari HR, Rad HG (2011). Nanotoxicology and nanoparticle safety in biomedical designs. Int J Nanomed.

[30] Ashcroft FM (1999). Ion Channels and Disease.

[31] Aidley DJ, Stanfield PR (1996). Ion channels. Molecules in action.

[32] Bezanilla F (2005). Voltage-gated ion channels. IEEE Trans Nanobiosci.

[33] Ide T, Aoki T, Takeuchi Y, Yanagida T (2006). Lysenin forms a voltage-dependent channel in artificial lipid bilayer membranes. Biochem Biophys Res Commun.

[34] Kiyokawa E, Makino A, Ishii K, Otsuka N, Yamaji-Hasegawa A, Kobayashi T (2004). Recognition of sphingomyelin by lysenin and lysenin-related proteins. Biochemistry.

[35] Kwiatkowska K, Hordejuk R, Szymczyk P, Kulma M, Abdel-Shakor A-B, Plucienniczak A, Dolowy K, Szewczyk A, Sobota A (2007). Lysenin-His, a sphingomyelin-recognizing toxin, requires tryptophan 20 for cation-selective channel assembly but not for membrane binding. Mol Membr Biol.

[36] Bokori-Brown M, Martin TG, Naylor CE, Basak AK, Titball RW, Savva CG (2016). Cryo-EM structure of lysenin pore elucidates membrane insertion by an aerolysin family protein. Nat Commun.

[37] Podobnik M, Savory P, Rojko N, Kisovec M, Wood N, Hambley R, Pugh J, Wallace EJ, McNeill L, Bruce M (2016). Crystal structure of an invertebrate cytolysin pore reveals unique properties and mechanism of assembly. Nat Commun.

[38] Bruhn H, Winkelmann J, Andersen C, Andra J, Leippe M (2006). Dissection of the mechanisms of cytolytic and antibacterial activity of lysenin, a defence protein of the annelid *Eisenia fetida*. Dev Comp Immunol.

[39] Fologea D, Krueger E, Mazur YI, Stith C, Okuyama Y, Henry R, Salamo GJ (2011). Bi-stability, hysteresis, and memory of voltage-gated lysenin channels. Biochim Biophys Acta Biomembr.

[40] Fologea D, Krueger E, Lee R, Naglak M, Mazur Y, Henry R, Salamo G (2010). Controlled gating of lysenin pores. Biophys Chem.

[41] Fologea D, Al Faori R, Krueger E, Mazur YI, Kern M, Williams M, Mortazavi A, Henry R, Salamo GJ (2011). Potential analytical applications of lysenin channels for detection of multivalent ions. Anal Bioanal Chem.

[42] Fologea D, Krueger E, Al Faori R, Lee R, Mazur YI, Henry R, Arnold M, Salamo GJ (2010). Multivalent ions control the transport through lysenin channels. Biophys Chem.

[43] Alanko GA, Thurber A, Hanna CB, Punnoose A (2012). Size, surface structure, and doping effects on ferromagnetism in SnO_2_. J Appl Phys.

[44] Hanley C, Thurber A, Hanna C, Punnoose A, Zhang JH, Wingett DG (2009). The influences of cell type and ZnO nanoparticle size on immune cell cytotoxicity and cytokine induction. Nanoscale Res Lett.

[45] Shrestha N, Bryant SL, Thomas C, Richtsmeier D, Pu X, Tinker J, Fologea D (2017). Stochastic sensing of angiotensin II with lysenin channels. Sci Rep.

[46] Bryant S, Shrestha N, Carnig P, Kosydar S, Belzeski P, Hanna C, Fologea D (2016). Purinergic control of lysenin’s transport and voltage-gating properties. Purinergic Signal.

[47] Montal M, Mueller P (1972). Formation of bimolecular membranes from lipid monolayers and a Study of their Electrical Properties. Proc Natl Acad Sci USA.

[48] Krueger E, Bryant S, Shrestha N, Clark T, Hanna C, Pink D, Fologea D (2016). Intramembrane congestion effects on lysenin channel voltage-induced gating. Eur Biophys J.

[49] De Colibus L, Sonnen AF, Morris KJ, Siebert CA, Abrusci P, Plitzko J, Hodnik V, Leippe M, Volpi E, Anderluh G, Gilbert RJ (2012). Structures of lysenin reveal a shared evolutionary origin for pore-forming proteins and its mode of sphingomyelin recognition. Structure.

[50] Misra SK, Dybowska A, Berhanu D, Luoma SN, Valsami-Jones E (2012). The complexity of nanoparticle dissolution and its importance in nanotoxicological studies. Sci Total Environ.

[51] Berg JM, Romoser A, Banerjee N, Zebda R, Sayes CM (2009). The relationship between pH and zeta potential of similar to 30 nm metal oxide nanoparticle suspensions relevant to in vitro toxicological evaluations. Nanotoxicology.

[52] Schwegmann H, Feitz AJ, Frimmel FH (2010). Influence of the zeta potential on the sorption and toxicity of iron oxide nanoparticles on *S. cerevisiae* and *E. coli*. J Colloid Interface Sci.

[53] Patil S, Sandberg A, Heckert E, Self W, Seal S (2007). Protein adsorption and cellular uptake of cerium oxide nanoparticles as a function of zeta potential. Biomaterials.

[54] Yang H, Liu C, Yang DF, Zhang HS, Xi ZG (2009). Comparative study of cytotoxicity, oxidative stress and genotoxicity induced by four typical nanomaterials: the role of particle size, shape and composition. J Appl Toxicol.

[55] den Hartog GJM, Qi SF, van Tilburg JHO, Koek GH, Bast A (2014). Superoxide anion radicals activate hepatic stellate cells after entry through chloride channels: a new target in liver fibrosis. Eur J Pharmacol.

[56] Sahoo N, Hoshi T, Heinemann SH (2014). Oxidative modulation of voltage-gated potassium channels. Antioxid Redox Signal.

[57] Yang Z, Liu ZW, Allaker RP, Reip P, Oxford J, Ahmad Z, Ren G (2010). A review of nanoparticle functionality and toxicity on the central nervous system. J R Soc Interface.

[58] Shah B, Kona S, Gilbertson TA, Nguyen KT (2011). Effects of poly-(lactide-co-glycolide) nanoparticles on electrophysiological properties of enteroendocrine cells. J Nanosci Nanotechnol.

[59] Liu ZW, Ren GG, Zhang T, Yang Z (2009). Action potential changes associated with the inhibitory effects on voltage-gated sodium current of hippocampal CA1 neurons by silver nanoparticles. Toxicology.

[60] Valdiglesias V, Costa C, Kilic G, Costa S, Pasaro E, Laffon B, Teixeira JP (2013). Neuronal cytotoxicity and genotoxicity induced by zinc oxide nanoparticles. Environ Int.

