# Supplementary material for: ZnO nanoparticles modulate the ionic transport and voltage regulation of lysenin nanochannels
Source: J Nanobiotechnology. 2017 Dec 16;15:90. doi: 10.1186/s12951-017-0327-9 (PMC5732404; doi:10.1186/s12951-017-0327-9)
Supplement: Supplementary file 1 — Additional file 1. Additional material that includes complementary figures of material characterization (Figs. S1–S6: XRD, TEM, DLS, XPS, and FTIR for ZnO and SnO2 NPs), changes in relative macroscopic conductance of lysenin channels in the presence of ZnO NPs and EDTA (Fig. S7), and conductance inhibition at high ionic strength (Fig. S8). [file 12951_2017_327_MOESM1_ESM.pdf]

Supplementary Material

ZnO Nanoparticles Modulate the Ionic Transport and Voltage Regulation of Lysenin Nanochannels

Sheenah L. Bryant[1,2], Josh E. Eixenberger[1,2], Steven Rossland[1,$], Holly Apsley[1,#], Connor Hoffmann[1,&], Nisha Shrestha[1,2], Michael McHugh[2], Alex Punnoose[1,2], Daniel Fologea[1,2,*]

[1] Department of Physics, Boise State University, Boise, ID 83725, USA

[2] Biomolecular Sciences Graduate Program, Boise State University, Boise, ID 83725, USA

Current addresses: [$] Dept. of Physics, University of Utah, Salt Lake City, UT 84112, USA;

[#] Dept. of Social Sciences, Yale-NUS College, Singapore 138610, Singapore;

[&] Dept. of Chemical and Biological Engineering, Montana State University, Bozeman, MT 59717, USA

E-mails: SheenahBryant@boisestate.edu, JoshEixenberger@boisestate.edu, coimhead1643@gmail.com, holly.apsley@u.yale-nus.edu.sg, connor.hoffmann@montana.edu, NishaShrestha@boisestate.edu, mikemchugh@u.boisestate.edu, apunnoos@boisestate.edu

[*]Corresponding author: DanielFologea@boisestate.edu

Supplementary Data for Material Characterization

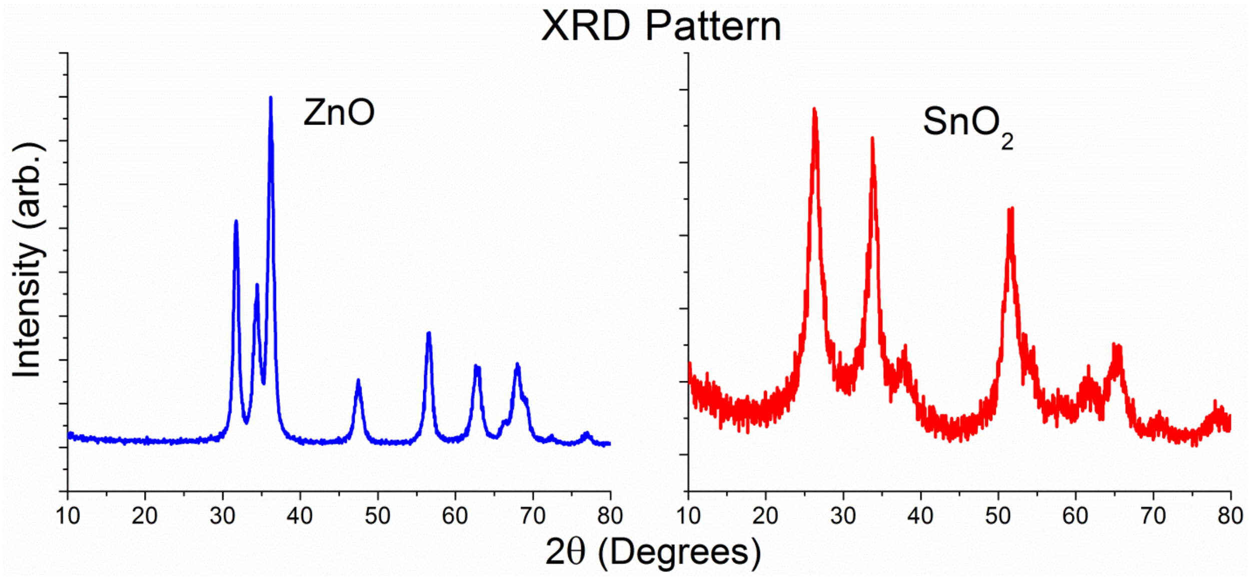

Figure S1. The XRD spectra of ZnO (left) and SnO_2 (right). For ZnO, XRD confirmed the hexagonal wurzite crystal structure with no alternate crystal phases detected. The average crystal size determined for ZnO was estimated at 8.3 nm. The XRD spectra of SnO_2 demonstrated the crystal phase obtained was cassiterite with no alternate phases present. The average crystal size for SnO_2 was determined to be 4.3 nm from the XRD spectra.

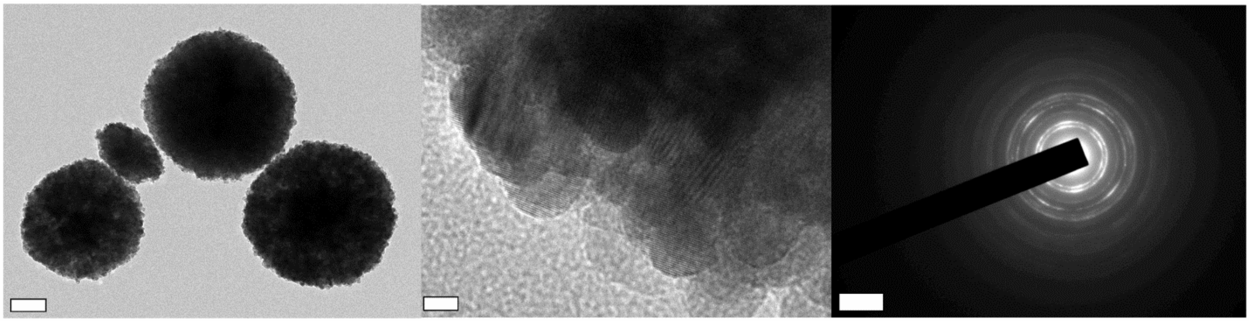

Figure S2. TEM images and electron diffraction pattern of ZnO nanoparticles. (Left; scale bar: 100 nm) The lower magnification image shows that the ZnO crystals aggregate to form larger spheres. (Middle; scale bar: 5 nm) Higher magnification images demonstrate that the large aggregates are comprised of small ZnO nanoparticles with an average size of ~10 nm. (Right; scale bar: 5 1/nm) The electron diffraction pattern obtained from the ZnO nanoparticle sample.

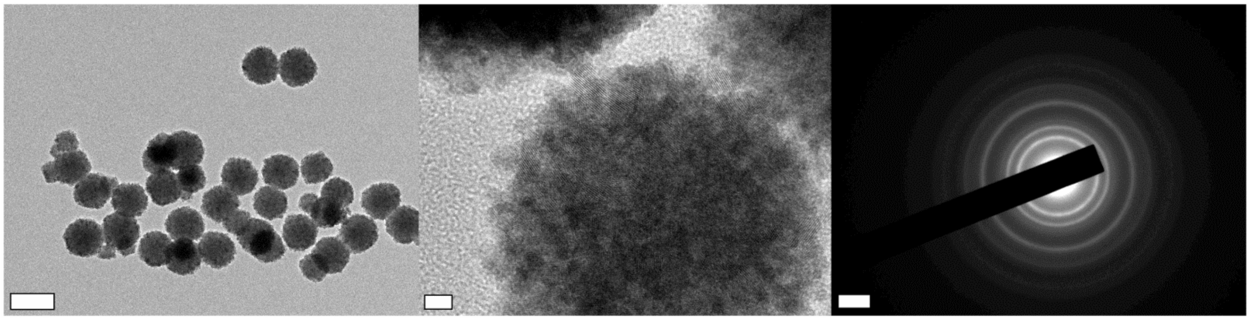

Figure S3. TEM images and electron diffraction pattern of SnO_2 nanoparticles. (Left; scale bar: 100 nm) The lower magnification image shows that SnO_2 nanoparticles also form larger aggregates which are generally smaller than the aggregates seen from the ZnO nanoparticles used. (Middle; scale bar: 5 nm) Higher magnification reveals that the aggregates formed are from small SnO_2 nanoparticles with an average size of ~5 nm. (Right; scale bar: 2 1/nm) The electron diffraction pattern obtained from the SnO_2 nanoparticle sample.

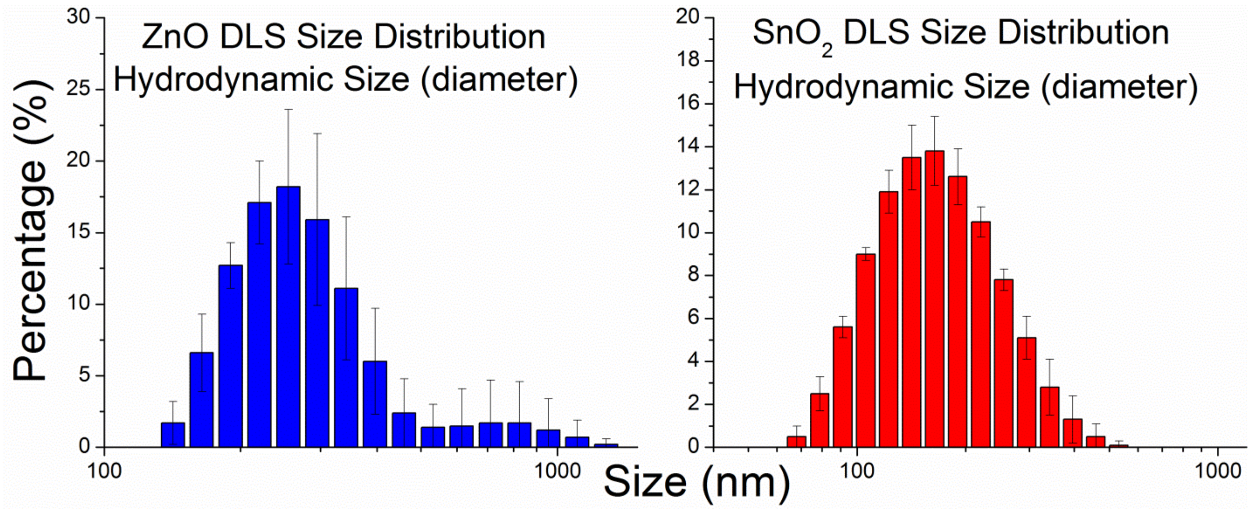

Figure S4. Hydrodynamic size distributions of ZnO (left) and SnO_2 (right) obtained from dynamic light scattering measurements. Hydrodynamic size distributions of SnO_2 demonstrate smaller average aggregates than ZnO, consistent with images obtained from TEM. The smaller particle size of SnO_2 suggests that these NPs would be better suited to inhibit conductance of lysenin channels by physical occlusion but this was not observed experimentally.

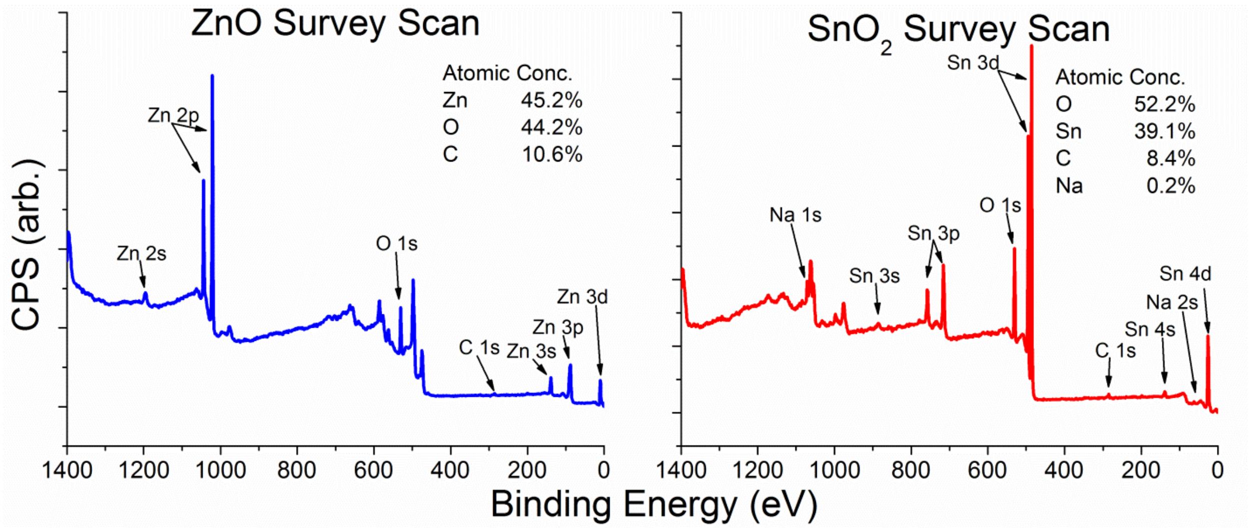

Figure S5. X-ray photoelectron spectroscopy survey spectra of ZnO (left) and SnO_2 (right) nanoparticles. Spectra demonstrate high sample purity, small retention of carbon species and sodium in the case of SnO_2, likely from carbon dioxide from atmospheric exposure and species retained from the chemical precursors.

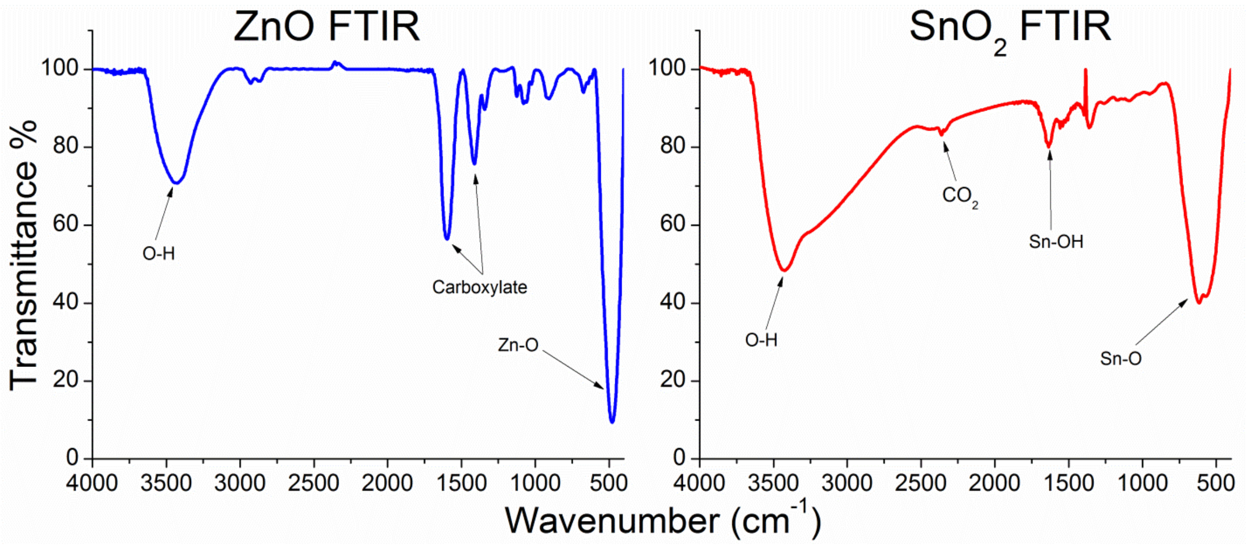

Figure S6. Fourier-transform infrared (FTIR) spectra of ZnO (left) and SnO_2 (right) nanoparticles. Spectra confirm retained species from chemical precursors and carbon dioxide, as well as demonstrate hydroxide species are present in both nanoparticles synthesized.

Supplementary data for conductance inhibition of lysenin channels by NPs

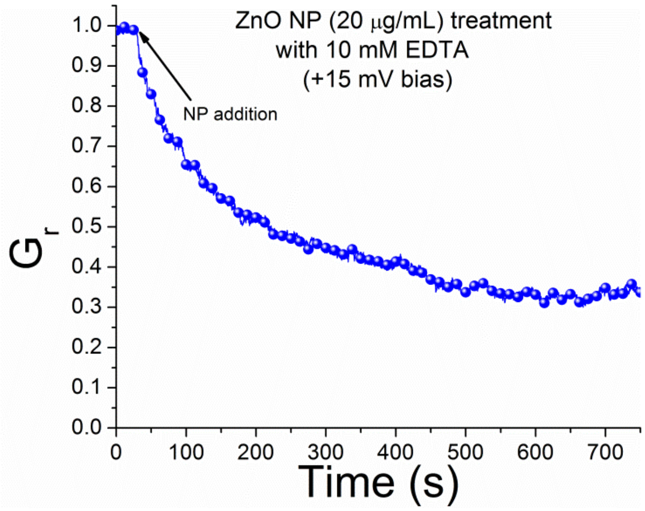

Figure S7. Interactions between ZnO nanoparticles and lysenin channels with EDTA (10 mM) present in the solution reservoirs. EDTA is a strong chelator of Zn^{2+} ions and thus prevents interactions between lysenin nanochannels and zinc ions. Nevertheless, ZnO nanoparticles elicited a strong conductance inhibition of lysenin channels, even with EDTA present, clearly demonstrating that inhibition is due to interactions between ZnO and lysenin, not free zinc ions.

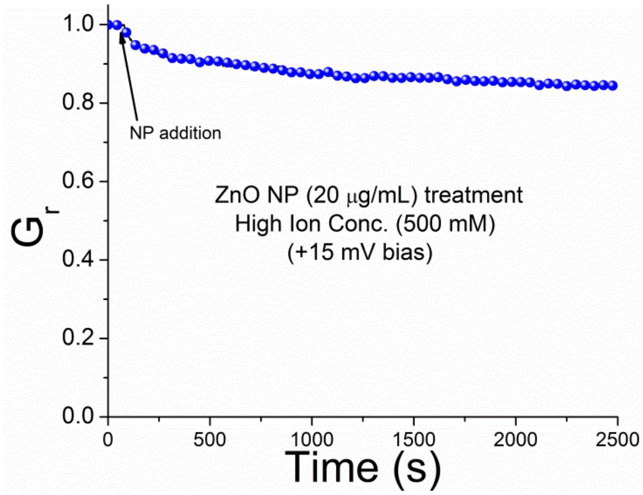

Figure S8. Ionic conductance of lysenin channels in the presence of ZnO nanoparticles while using a significantly higher electrolyte concentration in solution (500 mM). When compared with experiments under low electrolyte solutions (130 mM), the ZnO nanoparticles elicited a much weaker response. The reduction in conductance inhibition may be due to: 1) Debye screening effects that reduce the electrostatic interactions between ZnO nanoparticles and lysenin channels, preventing the initial binding event required for conductance inhibition and 2) increased aggregation of ZnO nanoparticles, which reduces interactions between lysenin channels and ZnO nanoparticles.
